# The rubber hand illusion is accompanied by a distributed reduction of alpha and beta power in the EEG

**DOI:** 10.1371/journal.pone.0271659

**Published:** 2022-07-29

**Authors:** Placido Sciortino, Christoph Kayser

**Affiliations:** Department of Cognitive Neuroscience, Faculty of Biology, Bielefeld University, Bielefeld, Germany; Georgia State University, UNITED STATES

## Abstract

Previous studies have reported correlates of bodily self-illusions such as the rubber hand in signatures of rhythmic brain activity. However, individual studies focused on specific variations of the rubber hand paradigm, used different experimental setups to induce this, or used different control conditions to isolate the neurophysiological signatures related to the illusory state, leaving the specificity of the reported illusion-signatures unclear. We here quantified correlates of the rubber hand illusion in EEG-derived oscillatory brain activity and asked two questions: which of the observed correlates are robust to the precise nature of the control conditions used as contrast for the illusory state, and whether such correlates emerge directly around the subjective illusion onset. To address these questions, we relied on two experimental configurations to induce the illusion, on different non-illusion conditions to isolate neurophysiological signatures of the illusory state, and we implemented an analysis directly focusing on the immediate moment of the illusion onset. Our results reveal a widespread suppression of alpha and beta-band activity associated with the illusory state in general, whereby the reduction of beta power prevailed around the immediate illusion onset. These results confirm previous reports of a suppression of alpha and beta rhythms during body illusions, but also highlight the difficulties to directly pinpoint the precise neurophysiological correlates of the illusory state.

## Introduction

The rubber hand illusion serves as an important paradigm to investigate the neurophysiological processes underlying multisensory body perception and the sense of body ownership. During the rubber hand illusion (RHI) the synchronous stimulation of an artificial hand in view and of participants’ occluded hand lets participants feel the rubber hand as becoming a part of their own body [1–3]. Despite the prominence of the rubber hand and related body illusions in the literature, the neurophysiological signatures of the participant’s illusory state remain debated.

One group of neuroimaging (EEG) studies has focused on signatures of rhythmic, i.e. oscillatory, brain activity. These studies focused on classical versions of the illusion involving a rubber hand [4, 5], on illusions in virtual reality environments [6], similar paradigms involving the embodiment of an artificial hand [7–9], or full-body illusions [10]. Collectively, these studies reported a reduction of alpha power over parietal and central electrodes during the illusory state [4–9], a reduction of beta band power over parietal regions [4, 5] or emphasized increases in gamma power [11] or changes in the inter-electrode synchronization in the gamma band [12–14]. Given that alpha and beta band activity have been linked to the overall excitability of the sensory-motor system [15–17], and given that beta activity has also been linked to changes in sensory-motor feedback and motor preparation [18–20], such spatially distributed alpha/beta signatures of the illusory state may reflect an increased activation of those sensory-motor regions linked to the illusory body ownership [5, 21–23].

Yet, given that individual studies used different experimental setups to induce the illusion, or used different control conditions to isolate those neurophysiological signatures related to the participant’s illusory state, the reliability and specificity of these results remain unclear. That is, the body of previous studies leaves it unclear whether the same electrode-frequency-wise pattern of oscillatory activity differentiates the illusory-state from non-illusory states regardless of the precise experimental conditions. Importantly, none of the previous studies directly probed whether the reported changes in alpha and or beta band activity indeed arise directly around the onset of the subjective illusion [11, 22]. In fact, most studies have quantified rhythmic brain activity over longer experimental time windows, e.g. by contrasting long illusion with long non-illusion trials. This leaves it unclear whether the reported changes in oscillatory activity are indeed directly related to the emergence of the illusory state. Alternatively, differences in time-averaged brain activity between separate experimental trials may also appear as a by-product of the illusion over the entire experimental trial, by attentional differences [5] or effects related to demand characteristics as debated recently [24–26].

We here address these questions in an explorative study [27] and quantify whether changes in EEG-derived oscillatory activity are systematically related to the illusory state during the RHI in two ways. First, by probing the robustness of such correlates to variations in the spatial plane in which the illusion is induced or to variations in the experimental control condition. For this we relied on two different experimental configurations to induce the illusion (having the rubber and besides or below the participant’s hand) and on different non-illusion conditions (involving the rubber hand placed in an unrealistic body position or only involving stimulation on the participants’ own hand) to implement statistical contrasts to isolate neurophysiological signatures of the participant’s illusory state. And second, we asked whether such correlates emerge directly around the subjective illusion onset. For this we contrasted data epochs immediately prior to and subsequent to the illusion onset. Each question was addressed using an unbiased approach in which we tested for significant effects over all electrode-frequency combinations, and in addition, using a region of interest (ROI) based approach. The latter serves as confirmatory analysis in which we focused specifically on the alpha and beta power over central and parietal electrodes, which have previously been implied in the RHI and related sensorimotor processes [4–6, 10].

## Materials and methods

The data analysed here was obtained during a previous study, where we report the behavioural data and an analysis of evoked responses [28].

### Participants and experimental procedures

Participants were informed about the details of the study and provided written informed consent prior to participation. All procedures were approved by the Ethics Committee of Bielefeld University. Experiments took place in a darkened and electrically shielded room (Ebox, Desone, Germany). To ensure that participants in the main study were indeed able to feel and report the rubber hand illusion we conducted a pre-screening. For this, a group of 54 participants was invited, none of whom reported having participated in a study on body illusions previously. The screening session included the two Illusion conditions and the two Incongruent conditions, each presented in a pseudo-random order. Details of the experimental conditions are provided below. Note that in the following we use the uppercase term Illusion to refer to the experimental condition and the lowercase term illusion for the general phenomenon. Participants were not informed on the possible behavioural or phenomenological outcomes of the single conditions, the nature of the different Illusion and Control condition and the nature of the visuo-tactile stimulation. To determine whether and when participants felt the illusion we capitalized on the key item from the rubber-hand questionnaire [2] and instructed participants to press a key on a computer keyboard when they were “feeling the rubber hand as belonging to their body”. Participants made these responses using their right hand [5]. For the main experiment, we included only participants from the screening session who had reported feeling the illusion in both Illusion conditions and had not reported feeling the illusion in any Incongruent condition, as indicated by the respective button press responses, and who responded to the standard rubber hand questionnaire [2] with a mean positive score for the Illusion questions and a mean negative score for the control questions in the Illusion conditions.

A total 24 participants were included in the main study. During the main study, the five conditions described below were each repeated 4 times, with trials administered in a pseudo random order for each participant. Each trial lasted for 3 minutes of visuo-tactile stimulation (180 stimulation events). We determined the onset of the subjective illusion as the time of the button press made by the participant on each trial. Administering the rubber hand questionnaire multiple times per experiment is unlikely to yield sensitive results, and hence we relied on the button press response as a test of the main item of the questionnaire pertaining to the embodiment of the rubber hand. We also administered the full questionnaire at the end of the first repeat of each of the two Illusion conditions (see [28] for details). The average scores for illusion (Illusion hand next: 2.38 ± 0.53, mean ± s.e.m.; Illusion hand under: 2.61±0.53) and control statements (hand next: -1.47 ± 0.76; hand under: -1.56 ± 0.80) confirmed that participants were experiencing the subjective illusory state as known from the literature. One participant had to be excluded from the main study as this participant reported feeling the illusion also during Incongruent trials, and one participant was excluded due to reporting the illusion for only one of the two Illusion conditions.

### Experimental conditions

The experimental conditions were designed to induce the RHI across either horizontal or vertical arrangements of the participant’s own and the rubber hand. They also comprised two different non-illusion conditions that could be used as contrast to isolate neurophysiological signatures of the illusory state, allowing us to probe the robustness of putative signatures of the illusory state across different comparisons of illusion and non-illusion conditions. Further rationale for the precise choice of conditions is provided after their description in the following.

Participants sat in front of a one compartment, open-ended box placed on a two-storey wooden platform with a rubber hand in front of them. The illusion was induced based on an automated procedure using the repetitive synchronous visuo-tactile stimulation controlled via Matlab and two Arduino Uno prototyping platforms, similar as previous studies [5]. A white light-emitting diode (LED; Seeedstudio, 10 mm diameter) was used for visual stimulation near the rubber hand and a vibration motor (Grove: Vibration motor, Seeedstudio) was placed below the left index fingertip of the participant and delivered the tactile stimulation. A copy of the stimulation signals was sent to the analogue input of the EEG system to ensure the precise alignment of the visuo-tactile stimulation events with the EEG data. The length of each visuo-tactile stimulation pulse was 100 ms and the interstimulus interval was 900 ms. The choice of the stimulus duration was based on pilot tests (n = 6), in which we varied the ISI between 300, 600 or 900 ms and based on our previous work [5]. During the pilot test the 900 ms ISI yielded the highest fraction of illusory states.

**Fig 1:**
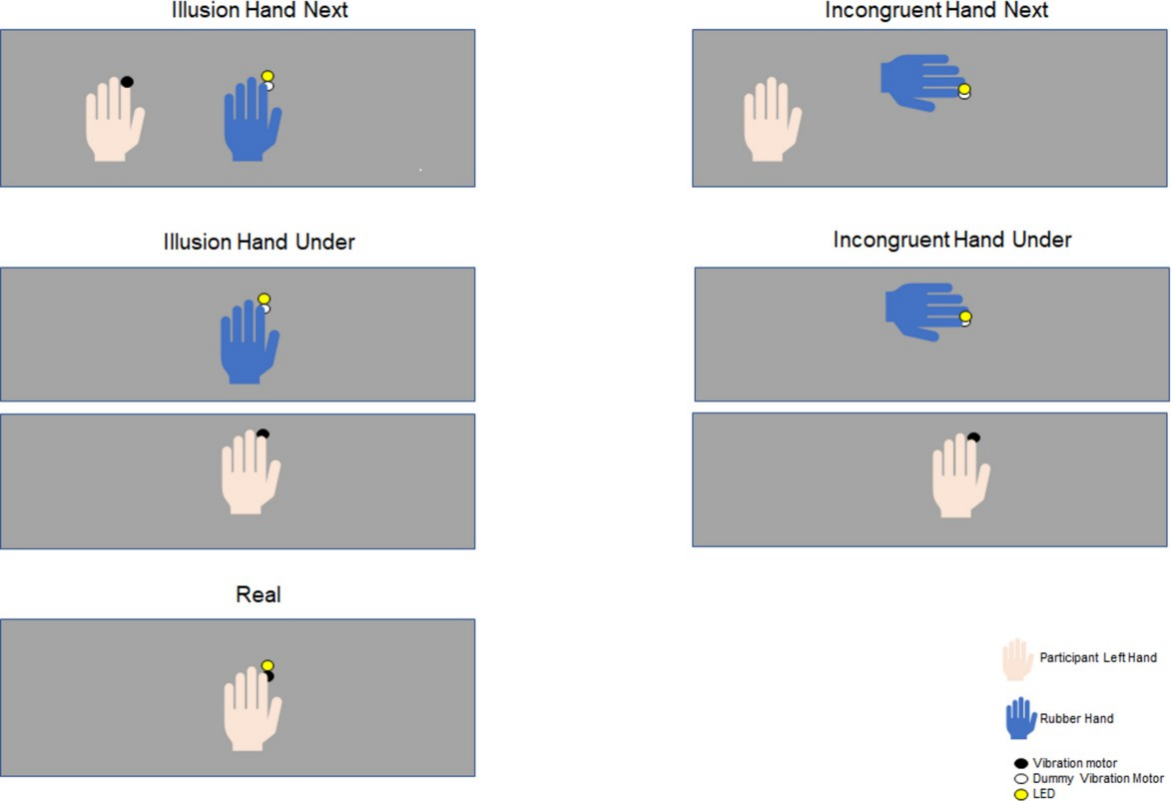
Schematic of the five experimental conditions. The two Illusion conditions were defined by the position of the participant’s own hand relative to the rubber hand in either the vertical or horizontal plane. Each Illusion condition was paired with its respective Incongruent control condition, derived by tilting the rubber hand by 90 degrees. In the Real condition the visuo- tactile stimulation was delivered on the participant’s hand and the rubber hand was absent.

The main experiment comprised five experimental conditions (two Illusion conditions, two Incongruent control conditions, and one Real control condition; Fig 1), that each control for a different facet of the illusory state (see also the Discussion in [28]). The two Illusion conditions differed in the spatial arrangement of the real and rubber hands (either side by side or below each other) and were named “Illusion hand next condition” and “Illusion hand under condition”. During the Illusion hand next condition, a life-like rubber hand model (for men: a silicon cosmetic glove, model 102LS, for women: model 102LS, ORTHO-REHA Neuhof GmbH) was placed in front of the participants in an anatomically congruent position, as typical in studies on the RHI. The left index finger of the rubber hand was placed on a dummy vibration motor and the LED was placed above the dummy motor below the rubber hand. Participant’s left hand was covered with a blanket and positioned 10 cm to the left from the rubber hand in the horizontal plane. The tip of the participant’s hidden index finger was positioned on the vibration motor. The Illusion hand next was contrasted with an Incongruent hand next condition, in which the rubber hand was placed in front of the participant but at a 90° angle, hence in an unrealistic body position. During the Illusion hand under condition, the rubber hand was also placed in front of the participant but the participant’s real hand was placed 10 cm under the RH in the vertical plane, in the lower panel of the platform and covered with a blanket. Apart from the relative hand position, the set-up was the same as in the hand next condition. The Illusion hand under condition was paired with an Incongruent hand under condition, in which the rubber hand was placed in an anatomically implausible position, tilted by 90°. Lastly, we used the Real condition as a further control. In this condition the rubber hand was absent and the visuo-tactile stimulation occurred on the participant’s real hand. In this condition the LED was positioned 5mm above the tactile stimulation on the index finger of the participant’s left hand.

We used the Incongruent condition as control because placing the rubber hand in an unrealistic posture is effective in prohibiting the illusory feeling [3, 5, 22, 29–34]. Some previous studies have used a non-illusion condition in which the rubber hand and the participant’s hand are stimulated temporally asynchronously. However, contrasting signatures of rhythmic brain activity across conditions involving different temporal patterns of sensory stimulation can be misleading, prompting us to use control conditions that rely on the same pattern of sensory stimulation as the Illusion condition. Finally, the Real condition allowed us to probe the differences between the natural embodiment of the real hand versus the embodiment of an artificial hand (the Illusion condition), a dimension that cannot be investigated by the Incongruent condition or by the asynchronous stimulation of a bodily-aligned RH used in other previous studies [5, 35, 36]. That is, in both the Illusion and the Real condition the hand in sight is embodied, but the nature of the hand is different. In addition, the spatial pattern of the illusion-inducing visual-tactile stimuli differs between these two conditions, as they were presented spatially coincident in the Real condition but spatially separated in the Illusion condition, a factor that was not manipulated in the comparison of Illusion and Incongruent conditions.

### EEG recording and pre-processing

The EEG signals were acquired using a 128 channel BioSemi system with Ag-AgCl electrodes mounted on an elastic cap (BioSemi, B.V., Netherlands). Electrode offsets were kept below 25 mV and the acquisition rate was 1028 Hz. The electrooculogram (EOG) was recorded using 4 additional electrodes placed at the outer canthi and below participants’ eyes. The data were analysed using Matlab (The MathWorks Inc., Natick, MA, USA) and the FieldTrip toolbox [37]. The data were band-pass filtered between 0.6 Hz and 90 Hz and resampled to 200 Hz as in previous work for further processing [38, 39] Denoising was implemented using ICA and components reflecting muscular artefacts, eye blinks, eye movements as well as poor electrode contacts were identified based on recommendations in the literature and confirmed based on visual inspection [40, 41]. We removed an average of 15.0 ± 1 (mean ± s.e.m.) components per participant. Then, the data were epoched around the visuo-tactile stimulation events (-400 ms to + 400ms around stimulation onset), of which there were 180 per trial. Epochs on which the signal exceeded 165 μV were removed. To render the data for the Illusion trials specific to the period when participants were experiencing the illusory state, we selected for the Illusion trials only those epochs after the time point of the subjective illusion onset (i.e. the button press response). As reported previously, the illusion onset times were 34 ± 5.9 s (mean ± SD) and 51 ± 7.7 s for the hand under and hand next conditions [28]. As this effectively removes early epochs in each 3-minute trial, we applied a similar selection to the other conditions: for these we retained only those epochs after the participant-specific median reaction time obtained from all Illusion trials. The average number of epochs retained for both Illusion conditions per participant were 897 ± 51 (mean ± s.e.m. across participants), for the Incongruent conditions 845 ± 56, and for the Real condition 434 ± 33 epochs.

### EEG analysis of oscillatory brain activity

The time-averaged power in different frequency bands was extracted in Fieldtrip using discrete prolate spheroidal multi-tapers. We computed the time and epoch-averaged power at frequencies between 5 and 49 Hz (with 1 Hz spacing below 20 Hz, and 2 Hz spacing above, using 2 Hz smoothing and padding the epochs to the next power of 2). Power estimates were obtained for each electrode separately. Depending on the specific analysis, we either combined all Illusion epochs and all Incongruent epochs respectively, or we derived the power for Illusion or Incongruent conditions separately for each hand position. We then contrasted Illusion, Incongruent and Real epochs (see Statistics below).

In a separate analysis we quantified the change in the power around the subjective illusion onset. For this, we derived the power in two shorter time windows: one comprised the 10 epochs prior to the button press in Illusion trials (from -12 to -2 relative to the epoch containing the button press) and one comprised the 10 epochs after this (+2 to +12). We avoided artefacts potentially arising from the button press itself by excluding the 4 epochs immediately around the button press [42, 43]. The duration of this window (10 epochs correspond effectively to 10 s) was chosen as a compromise to retain sufficient epochs to obtain reliable estimates of power (10 epochs x 4 repeats x 2 Illusion conditions) while remaining sufficiently specific around the reported illusion onset and was constrained by the shortest illusion onset times on some trials, which were around 13 s. Because differences in power between the time windows prior and after the event of interest could in principle also arise from other factors than the illusion, such as a general change in oscillatory power during the progression of the trial, we implemented two control analyses: these were obtained by contrasting the power between pairs of time windows chosen such that one would not expect a change in power related to the illusion onset. First, we derived the power in two time windows later in the same Illusion trials. Here we centred two-time windows around the time point 160 s, which was chosen to be longer than the latest reported illusion onset on any individual trial. Second, we extracted time windows around the time point of illusion onset (within the 3-minute trial) using the data from the Incongruent trials. In this comparison no effect attributable to the illusion onset should be present, while any overall change in power within the 3-minute trial should be the same as during the Illusion trial.

**Fig 2:**
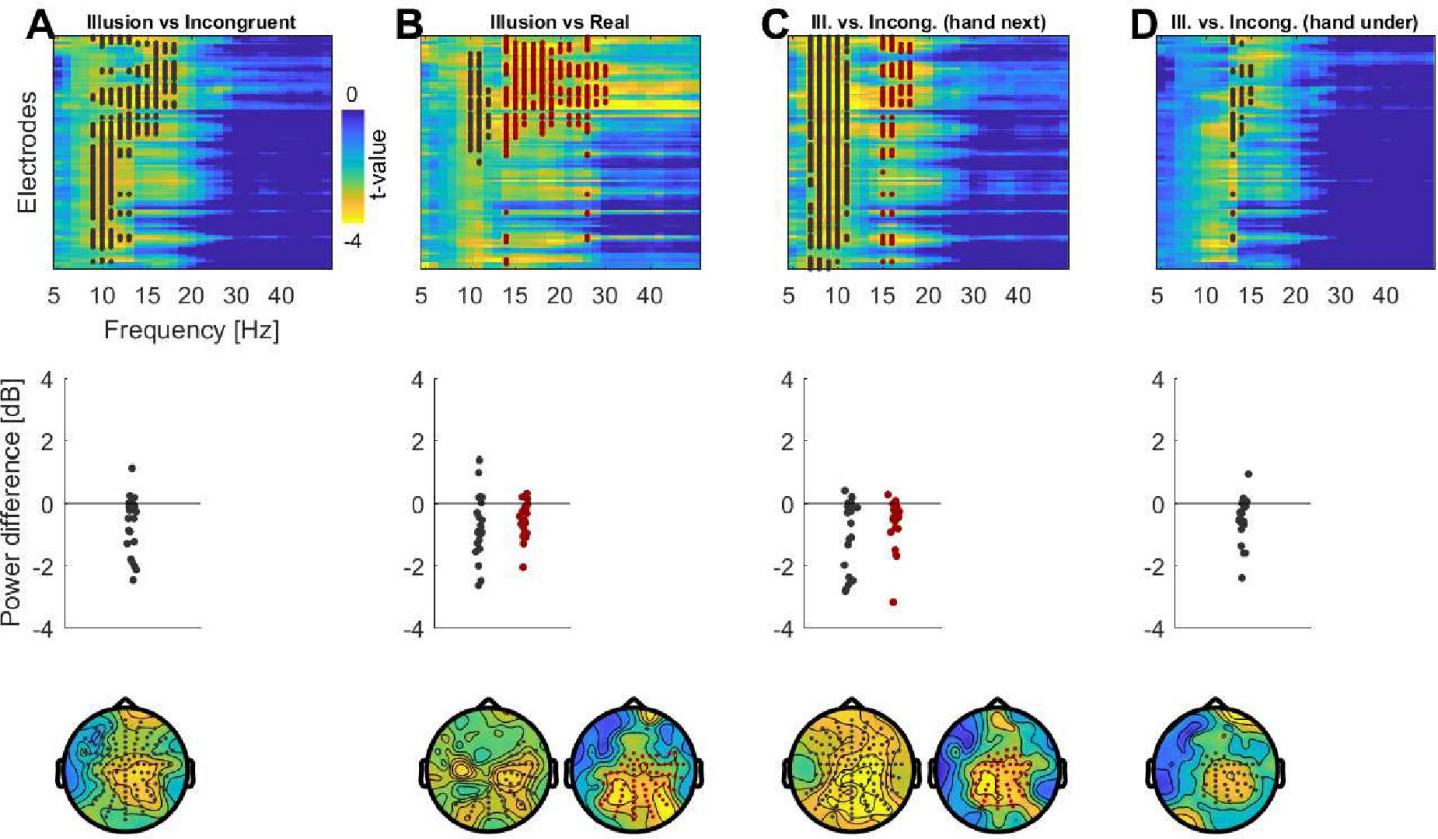
Differences in spectral power between conditions. A, B) Contrasting spectral power between Illusion and Incongruent conditions and between Illusion and the Real condition reveals a significant suppression in alpha and beta bands. The upper panel displays the electrode and frequency wise t-maps for a difference between conditions (two-sided t-test, showing Illusion minus Incongruent/Real). The middle panel shows the participant-wise power differences for the significant cluster(s). The lower panel shows the scalp topographies at the frequencies of the significant cluster(s). C, D) Results for the contrast between Illusion and Incongruent conditions separately for the ‘hand next’ and ‘hand under’ arrangements.

**Fig 3:**
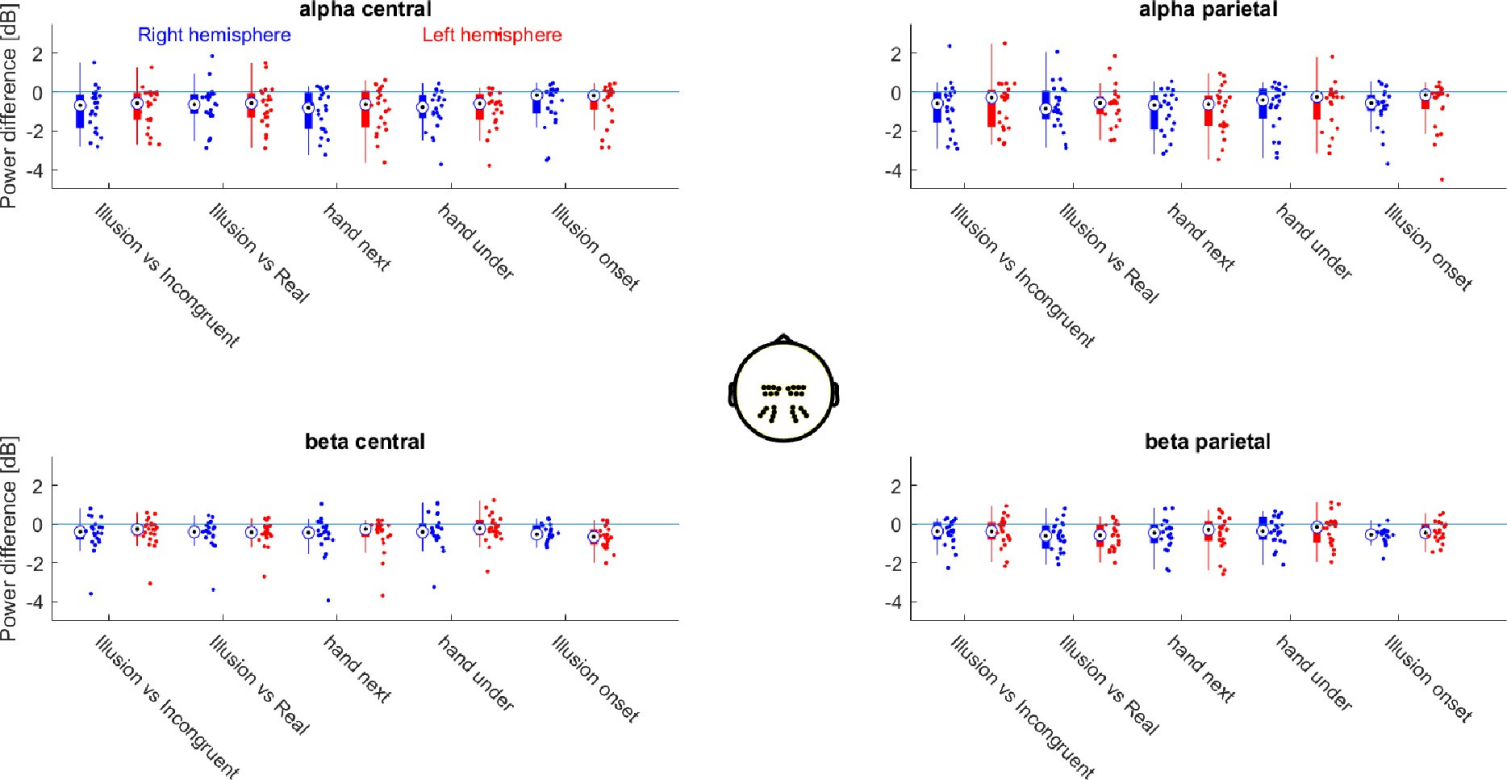
Hemisphere-specific differences in alpha and beta power over central and parietal regions of interest. Central panel shows the 4 ROIs, defined respectively by left and right central and parietal electrodes. Boxplots show the participant-wise power differences for the individual contrasts indicated, ROI and frequency band. Dots indicate individual participants. ‘hand next’ and ‘hand under’ refer to the comparison of Illusion and Incongruent for each hand arrangement separately. Ill: Illusion condition, Inc: Incongruent condition.

We also implemented a region of interest (ROI) based analysis. This was done for two reasons: first, to allow a confirmation of the results obtained using the unbiased approach, and second to allow a comparison to previous studies. These have reported significant effects across electrodes covering premotor and parietal sites (4,6), frontocentral and parietooccipital sites (5) and medial sensorimotor and premotor cortices (10). Hence, we chose the ROIs to cover the significant effects from the unbiased approach (c.f. Fig 2) and those effects reported in previous work. Practically, the four ROIs were defined as follows (in terms of the BioSemi 128 EEG layout): a right and left central/sensorimotor ROI (right: ’B1’, ’B2’, ’B18’, ’B19’, ’B20’, ’B21’, ’B22’; left: ’D15’, ’D28’, ’D16’, ’D17’, ’D14’, ’D18’, ’D19’) and a right and left posterior/parietal ROI (right: ’A31’, ’A30’, ’A29’, ’A28’, ’B5’, ’B6’, ’B7’; left: ’A18’, ’A17’, ’A16’, ’A15’, ’A8’, ’A9’, ’A10’ (depicted in Fig 3, middle panel). We included separate right- and left-lateralized ROIs to be able to test for a potential lateralization of illusion-correlates. For each ROI we extracted the alpha (8-12Hz) and beta (15–23 Hz) power averaged over all electrodes in each ROI.

### Statistical testing

The comparisons of interest were between the group-level average power contrasted between conditions, such as between Illusion and Incongruent, or between Illusion and Real, or between the epochs prior to and those subsequent to the illusion onset. To test for such differences across electrodes and frequency bands we relied on a cluster-based permutation procedure [44]. Specifically, we computed electrode- and frequency-wise paired t-tests between conditions and thresholded these at a first-level threshold of p<0.01 (two-sided). Significant electrode-frequency bins were aggregated using the cluster-mass (using a minimal cluster-size of 3) and the cluster-wise statistics in the actual data were compared to a surrogate distribution obtained from 5000 randomizations. For each significant cluster we report the p-value, the cluster-mass as test-statistics, and as effect size we report Cohen’s D at the bin with maximal effect size. For the ROI-based analysis we contrasted conditions (or the strength of power reduction between left- and right-lateralized ROIs for the analysis on lateralization) using paired two-sided t-tests and derived the associated Bayes factors (BF) using the Bayes Factor toolbox in Matlab (DOI 10.5281/zenodo.4849568) whereby we relied on the Jeffreys-Zellner-Siow (JZS), as recommended [45]. Bayes factors reflect the strength of evidence either against the null hypothesis of no difference in mean power between conditions, or in favour of this null hypothesis [45, 46]. When interpreting the BF values, we refer to the nomenclature of Raftery [47]. Therefore, we interpreted BF between 1 and 3 as ‘weak’ evidence, BF between 3 and 20 as ‘positive’ evidence, between 20 and 150 as ‘strong’, and > 150 as ‘very strong’ evidence. As the present study presents an analysis of a dataset obtained previously [8], we did not plan the sample size *a priori* for these specific analyses. Still, we asked whether the sample of n = 22 would be sufficient to reproduce previous reports of changes in oscillatory activity associated with body-related illusions. From three studies we derived the effect sizes for the main contrast of interest from the numbers reported in the text, tables or figures (5–6, 10): theses yielded values of Cohen’s D of 0.5, 0.89 and 1.2 respectively. For a paired two-sided t-test with an alpha level of 0.01 and a power of 90% our sample size would be sufficient to yield a significant effect for two of these three previous studies.

## Results

### The illusory state is accompanied by a reduction of alpha and beta power

**Fig 4:**
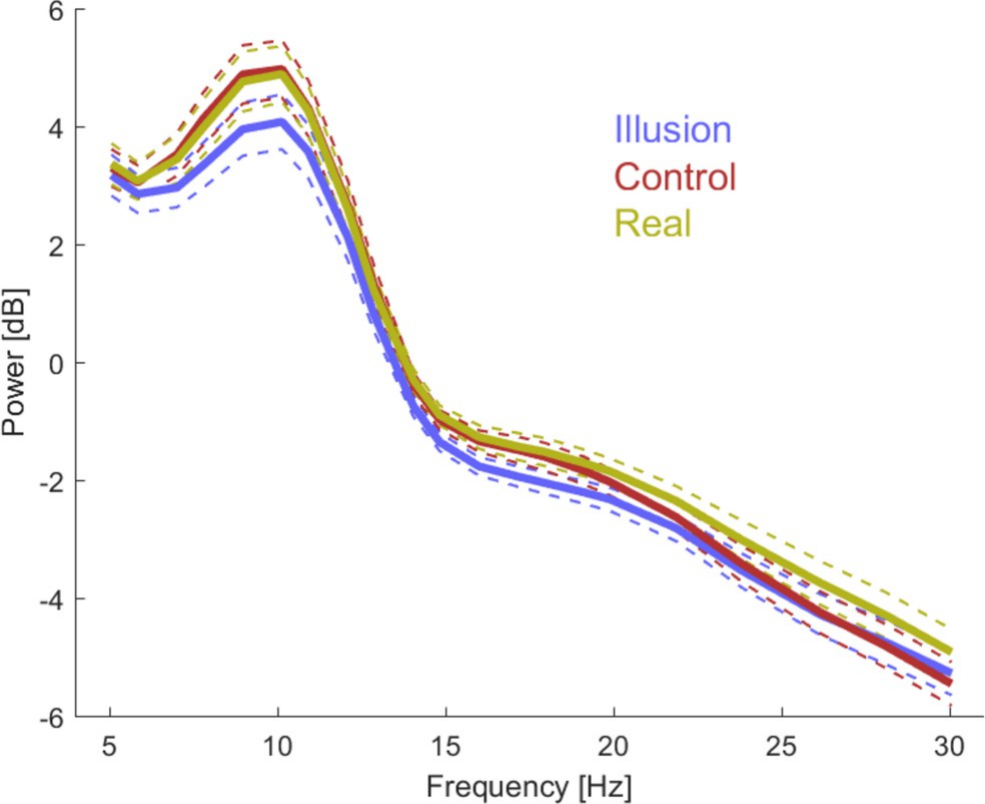
Overall spectral power for individual conditions. The graph illustrates the electrode and participant-averaged spectra for the Illusion, Incongruent (both averaged across hand positions) and the Real conditions. These suggest a reduced power during the Illusion condition compared to both control conditions in the alpha and beta bands. Thick lines indicate the mean and dashed lines the standard error across participants (n = 22).

The main focus was to compare strength (power) of rhythmic brain activity between the illusory state and the different control conditions. To illustrate the overall spectral power of the EEG signals, Fig 4 shows the participant- and electrode-averaged spectra for each condition. These suggest an illusion-related suppression in the alpha (about 8 to 12 Hz) and beta band (about 14 to 22 Hz) compared to both Control and Real conditions.

In a first approach we quantified statistical differences between Illusion and control conditions across electrodes and frequencies. For this we relied on cluster-based permutation procedures to test for differences in the group-average power (paired two-sided t-tests), which also controls for multiple comparisons along frequencies and electrodes. When combining both hand positions, this revealed a significantly reduced power during Illusion compared to Incongruent epochs around the alpha and beta bands (n = 22; p = 0.004, tsum = -1459, Cohen’s D = -0.93, 9.0–18.0 Hz; 111 electrodes, see Fig 2A). A similar result was obtained when contrasting Illusion and Incongruent epochs for each hand position separately: hand next arrangement (Fig 2C; Cluster 1: p = 0.001 Cohen’s D, tsum = -1908, Cohen’s = D -0.98, 7.0–10.9Hz, 122 electrodes; Cluster 2: p = 0.008, tsum = -465, Cohen’s D = -1.00, 14.8–18.0 Hz, 51 electrodes) and hand under arrangement (Fig 2D; p = 0.018, tsum = -243, Cohen’s D = -0.88, 12.9–14.1 Hz, 44 electrodes). Probing a different contrast to isolate correlates of the illusory state, we then compared the Illusion to the Real condition. This again revealed a significantly reduced power during the Illusion epochs (Fig 2B; Cluster 1: p = 0.012, tsum = -355, Cohen’s D = -0.83, 10–12 Hz, 50 electrodes; Cluster 2: p = 0.009, tsum = -1287, Cohen’s D = -1.13, 14.1–30.0 Hz, 71 electrodes).

### A reduction of beta power emerges directly around illusion onset

**Fig 5:**
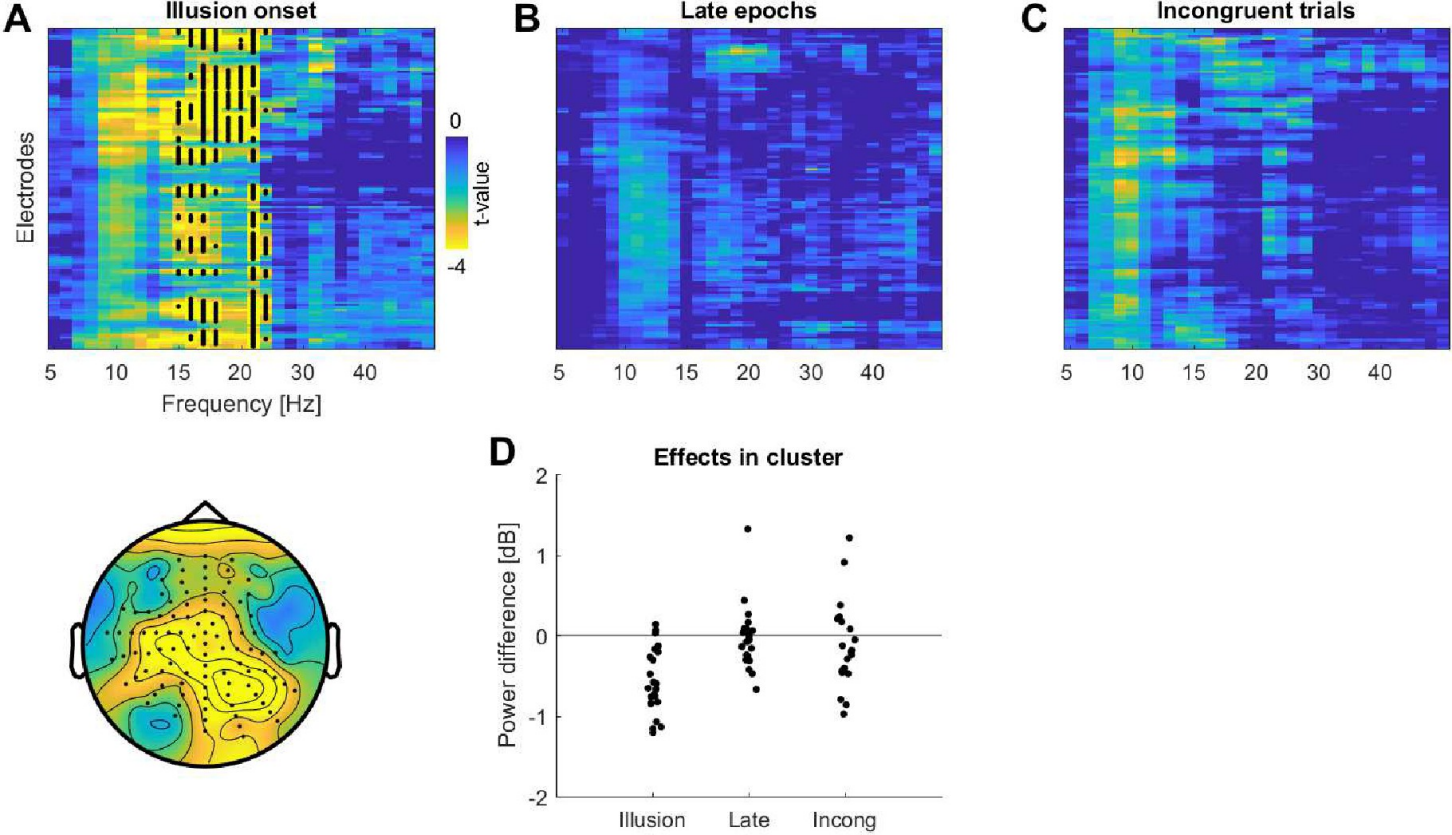
Changes in spectral power around illusion onset and at control time points. **A)** Contrast between the power between epochs immediately prior to the illusion onset and the epochs immediately after the onset. **B)** Contrast between the epochs taken from a later period of the Illusion condition. **C)** Contrast between epochs in the Incongruent condition prior and subsequent to the times of the illusion onset in the Illusion trials. Panels A-C show the electrode and frequency wise t-maps for a difference between epochs (two-sided t-test, showing post illusion onset minus prior to illusion epochs). The lower panel in A) shows the scalp topography at the frequencies of the significant cluster. **D)** Shows the participant-wise power differences between conditions for the electrodes and frequencies derived from the significant cluster in A.

In a second analysis we asked whether this reduction in power specifically emerges around the time of the illusion onset. For this we contrasted the power between data epochs immediately prior to the reported illusion onset with epochs immediately after this (see Materials and Methods). This revealed a significant cluster in which the power was reduced following the illusion onset in the beta band (n = 22; p<0.001, tsum = -1691, Cohen’s D = -1.68, 14.8–23.8 Hz, 98 electrodes; Fig 5A). To determine whether this reduction of beta power was indeed specific to the illusion onset, we implemented two control analyses. In one we contrasted epochs taken from a later period of the illusion trial: this revealed no significant cluster (at p<0.05; Fig 5B). In another control analysis, we used the same absolute time points of the illusion onset in the 3 minute trials, but quantified the power in Incongruent trials devoid of the illusory state. Again, this revealed no significant cluster (at p<0.05; Fig 5C).

As the absence of a significant effect is not proof of absence of an effect, we further investigated the time- and electrode-averaged power in the cluster obtained around illusion onset (Fig 5D). This confirmed, as expected, ‘very strong’ evidence for a significant difference in power around illusion onset in the Illusion condition (t = -6.456, p<0.001, BF = 8964). Importantly, this provided positive evidence against a difference in power for the epochs late in the Illusion trial (t = -0.426, p = 0.67, BF = 0.2) and weak evidence for the Incongruent trial (t = -1.310, p = 0.20, BF = 0.5). We then quantified the illusion-related change in power (power after the time of interest minus that before). When contrasted between illusion onset and the two control time windows, this revealed ‘strong’ evidence for a larger power change around illusion onset compared to the late epochs in the Illusion trials (t = -4.12, p<0.001, BF = 68.1) and ‘positive’ evidence for the epochs from Incongruent trials (t = -3.12, p = 0.005, BF = 8.7). These results show that the reduction in beta power around the illusion onset is stronger compared to control conditions in which any change in power could not arise from the illusory state.

### Alpha and beta power are reduced over both central and parietal electrodes

We performed a second analysis specifically focusing on alpha and beta power over central and parietal electrodes. This allowed us to directly confirm that the power in both bands and over both ROIs was indeed reduced during the illusory state. Importantly, this allowed us to also answer the following questions that were not addressed above. First, whether a specific reduction in power (over a given ROI and band) is observed consistently in both arrangements of hands used to induce the illusion and between the different statistical contrasts used to isolate the illusion effect. If so, this would speak in favour of an effect that generalizes across statistical comparisons used to isolate an illusion correlate. Second, it allowed us to probe whether any reduction in power is comparable between hemispheres or whether this is possibly lateralized. The electrodes and frequencies of interest were determined based on the above results (e.g. the peaks in Fig 2A and 2B) and based on previous studies implying central or parietal activity in body illusions or sensorimotor processes [4–6, 10]. For the interpretation of these results we rely on the associated Bayes factors, given the larger number of comparisons performed.

For both bands and ROI’s we found ‘positive or ‘strong’ evidence for a reduction of power in the Illusion compared to the Incongruent or Real conditions when combining the data across hand arrangements (see Table 1 “ROIs”). Comparing the reduction in power between the two contrasts (Illusion vs. Incongruent and Illusion vs. Real) revealed no or ‘weak’ evidence, suggesting a comparable reduction in power between contrasts (see Table 1, “Pairwise comparison”). When comparing Illusion and Incongruent conditions for each hand arrangement separately we found ‘positive’ or ‘strong’ evidence for a reduction in power during the hand next arrangement, while for the hand under arrangement we found ‘positive’ evidence only for central alpha. Comparing the reduction in power (Illusion minus Incongruent) between hand arrangements again provided ‘moderate’ evidence for no difference between hand arrangements (see Table 1, “Pairwise comparison”). For the comparison of the data epochs prior to and subsequent the illusion onset, this ROI based analysis revealed ‘very strong’ evidence for a power reduction in beta power and ‘moderate’ evidence for a reduction of alpha after illusion osnet. Comparing the reduction in power between this contrast and the comparison of Illusion vs. Incongruent epochs provided mixed and mostly ‘weak’ evidence. Together, these ROI-based results suggest that both alpha and beta power are reduced to a comparable degree in multiple comparisons designed to extract correlates of the illusory state, and this reduction in alpha and beta power is observed over both central and parietal electrodes.

**Table 1:** ROI-based results for central and parietal alpha and beta power.

***ROIs***												
	**Central Alpha**			**Central Beta**			**Parietal Alpha**			**Parietal Beta**		
	**BF**	**P Val**	**Tstat**	**BF**	**P Val**	**Tstat**	**BF**	**P Val**	**Tstat**	**BF**	**P Val**	**Tstat**
**Ill vs. Incon**	35,8	0,001	-3,8	2,9	0,020	-2,5	4,1	0,013	-2,7	8,8	0,005	-3,1
**Ill vs. Real**	10,6	0,004	-3,2	13,7	0,003	-3,4	7,9	0,006	-3,1	238	p<0.001	-4,7
**hand next**	56,5	0,001	-4,0	6,1	0,008	-2,9	64	0,001	-4,1	6,8	0,007	-3,0
**hand under**	4,0	0,013	-2,7	0,8	0,099	-1,7	0,5	0,221	-1,3	2,6	0,022	-2,5
**Illusion onset**	5,3	0,009	-2,9	1016	p<0.001	-5,4	8,7	0,005	-3,1	1078	p<0.001	-5,4
***Pairwise comparisons***												
	**Central Alpha**			**Central Beta**			**Parietal Alpha**			**Parietal Beta**		
	**BF**	**P Val**	**Tstat**	**BF**	**P Val**	**Tstat**	**BF**	**P Val**	**Tstat**	**BF**	**P Val**	**Tstat**
**Ill vs. Incon / Ill vs. Real**	-3,1	0,365	0,9	-3,8	0,565	-0,6	-4,5	0,899	0,1	-1,3	0,106	-1,7
**hand next / hand under**	-3,8	0,538	0,6	-3,3	0,420	-0,8	-4,5	0,988	0,0	-4,2	0,686	-0,4
**Ill vs. Incon/ Illusion Onset**	-3,6	0,500	0,7	1,1	0,066	1,9	-1,6	0,132	1,6	-2,6	0,278	1,1
***Lateralization***												
	**Central Alpha**			**Central Beta**			**Parietal Alpha**			**Parietal Beta**		
	**BF**	**P Val**	**Tstat**	**BF**	**P Val**	**Tstat**	**BF**	**P Val**	**Tstat**	**BF**	**P Val**	**Tstat**
**Ill vs. Incon**	0,3	0,39	-0,9	0,7	0,12	-1,6	0,6	0,13	-1,6	0,2	0,84	-0,2
**Ill vs. Real**	0,2	0,80	0,3	0,2	0,65	-0,5	0,4	0,28	-1,1	0,2	0,92	0,1
**hand next**	0,4	0,31	-1,0	0,3	0,47	-0,7	0,3	0,45	-0,8	0,2	0,78	-0,3
**hand under**	0,2	0,74	-0,3	1,1	0,06	-2,0	0,7	0,11	-1,7	0,2	0,97	0,0
**Illusion onset**	0,3	0,49	-0,7	26	0,001	3,7	0,3	0,47	-0,7	8,4	0,01	-3,1

For each comparison we report the p-value, the t-value and the Bayes Factor (BF). N = 22. The section ‘ROIs’ focuses on the reduction in power between Illusion-related and non-illusion epochs, with the data averaged across both hemispheres for each ROI and band. The section ‘Pairwise comparisons’ compares the reduction in power (Illusion–non illusion condition) between the contrasts indicated. The section ‘Lateralization’ compares the reduction in power for each contrast between the left and right hemispheres for each ROI and band (negative t-values indicate a stronger power reduction in the right hemisphere). ‘hand next’ and ‘hand under’ refer to the comparison of Illusion and Incongruent for each hand arrangement separately. Ill: Illusion condition, Inc: Incongruent condition.

Finally, we probed for a lateralization of the reduction in power during the illusion between hemispheres (see Table 1 “Lateralization”). For the analysis around the illusion onset we found ‘strong’ evidence for a lateralization of central beta and ‘positive’ evidence for a lateralization of parietal beta. For all the other combinations of bands and ROI’s we mostly obtained ‘positive’ evidence against a lateralization.

## Discussion

The aim of this study was to investigate the correlates of the RHI in oscillatory brain activity derived from human EEG recordings. In particular, we asked how robust such correlates are to variations in the non-illusion control conditions used as contrast for the illusory state, and whether such changes emerge directly around the onset of the subjective illusory state. Our results point to a spatially distributed reduction of alpha and beta band power associated with the illusory state, whereby the reduction of beta power was most prominently observed across all statistical contrasts that isolate the illusory state, including its onset.

### Spatially distributed changes in oscillatory power associated with the illusory state

Previous studies on the RHI differed in the precise experimental setup used to induce the illusion or the control conditions used as contrast for the illusory state ([30] for a review). This leaves it unclear whether the previous reports of a reduction in oscillatory power are specific to the precise nature of the experimental setting or the control conditions used. For example, the RHI can be induced when the participant’s hand is beside [5, 21, 48–50] or below the rubber hand [35, 51, 52]. Yet it remains unclear whether the same pattern of oscillatory activity differentiates the illusion from control conditions in both spatial configurations. To address this question we implemented two Illusion conditions that induced the illusion by separating the participant’s real and the rubber hand along either the horizontal or the vertical plane. In addition, we implemented non-illusion control conditions either using a misaligned rubber hand or in a condition in which the visuo-tactile stimulation occurred on the real hand in view. We then analysed the data both using an unbiased approach contrasting conditions across all electrodes and a wide range of frequencies and, in addition, using a ROI-based approach focusing specifically on the alpha and beta band. Both analyses converge to the main finding of a reduction of oscillatory alpha and beta band activity that differentiates the illusion from all non-illusion control conditions. This reduction in power emerged to a comparable degree and regardless of the precise arrangements of the real and rubber hands or the nature of the control condition and is largely bilateral. The only exception to this picture was the reduction of beta power around illusion onset, as discussed next.

Most previous studies on oscillatory activity during the RHI obtained the condition-specific power estimates by averaging over the entire experimental trials and compared these between illusion and control conditions presented in separate trials during the experiment. While this can give insights about differences in oscillatory activity between experimental conditions, such a comparison does not provide information on the potential changes that directly emerge around the onset of the subjective illusion. Addressing this question, we contrasted the oscillatory power between epochs prior to and immediately subsequent to the individual illusion onset. This revealed a reduction in beta power that was significantly larger around illusion onset compared to changes in power in two control analyses. When considered in the context of all statistical contrasts explored, this reduction in centro-parietal beta power was the most consistent effect observed, suggesting it as a prominent marker of the illusory state in oscillatory activity to be investigated in more detail in the future.

One potential limitation of the within-trial comparison relates to the decision process underlying the objective reports of the illusory feeling. This comprises the accumulation of relevant sensory evidence, motor preparation and motor execution and also changes in task-set once a response has been given. In addition, there may have been individual variations in the decision criteria, as some participants may have reported the onset of the illusion only when they were very confident about this, and hence later than the actual onset, while others may have reported the onset much earlier, and even when not yet fully confident. Hence, the objective reports about the illusion onset do not necessarily indicate the precise onset of the subjective illusory state and the observed reduction of alpha and beta power around the reported illusion onset may reflect both the subjective illusion strength and participants decision criteria. However, the statistical comparison between the distinct Illusion and Condition trials mitigates against such decision effects, as it relies on brain activity obtained only after the illusion onset and averaged over many seconds or even minutes. This analysis should effectively reduce the influence of potential decision processes and individual decision criteria. In addition, we mitigated for any influence of motor preparation and execution by excluding data epochs immediately around the reported illusion onset. Overall, we believe that the collective results from multiple statistical comparisons suggest a reduction of alpha and beta power that is independent of the decision process when reporting the illusory state.

### What sensory or cognitive processes are reflected in the changes of alpha and beta power?

The present results converge with a body of previous studies that has revealed correlates of the RHI or related body illusions in rhythmic brain activity in the alpha and beta bands [4–10, 12–14]. Still, changes in alpha and beta band activity have been associated with a wide range of phenomena, which makes the interpretation of the distributed reduction in power not straightforward, as we discuss in the following. According to one prominent model, the RHI emerges as a result of the discrepant visual and tactile signals that are integrated in multisensory brain regions, giving rise to an altered perception of the body and of the sensory stimuli driving the illusion [1, 3, 22, 53–55]. In this context reduced alpha band activity during the illusion may be related to an enhanced processing demand in the somatosensory system in response to the discrepant multisensory stimuli [15–17, 19, 20]. In agreement with this proposal, changes in alpha power have been reported in various sensorimotor processes including the perception of body parts [56], the embodiment process itself [4, 5], or perspective-changes on human touch [57].

An alternative suggestion holds that the behavioural reports obtained during RHI paradigm, may, at least in part, result from demands implicitly imposed by the experimental setting. In response to such demands, top-down mechanisms may govern participants behaviour or even directly shape low-level sensory processes [24, 58–62]. This idea is consistent with a cognitive account of the RHI, which relates the reported illusory feeling to imaginative processes [58, 63, 64] that may act solely or in addition to bottom-up multisensory integration. Such a cognitive account is supported by the overlapping mechanisms of body ownership and motor imagery [6], and by the suggestion that the some aspects of participants behavioural reports obtained in rubber hand paradigms can possibly be induced by differences in demand characteristics rather than differences in the sensory stimulation itself [58–62]. Along this line, previous work argued that the influence of demand characteristics on the RHI may be mediated by traits such as hypnotisability [24, 58–62] and electrophysiological studies on hypnosis have provided some evidence for a link between alpha and beta rhythms and hypnotisability [65–68]. We did not quantify traits such as hypnotisability in our participants and hence we can’t strictly rule out a contribution of this to the reported results. However, the contrast of brain activity within the same Illusion trial should reduce potential influences of such demand characteristics. Yet, given the diverging opinions on the role of demand characteristics [25, 58–62, 69] further work is required to disentangle the neurophysiological correlates of top down phenomenological aspects of the illusory state from the sensory-level ones [64].

Parietal alpha band activity has also been linked to visual spatial attention, with reduced alpha power reflecting a stronger attentional engagement [70–72]. One previous study on the rubber hand illusion has suggested that the illusory state a may also be accompanied by a change in attention [5], and predicted that such a change in attention should be more clearly visible in the ‘hand next’ arrangement, in which the participant’s real and the rubber hand are separated along the horizontal plane. In the present data the reduction in alpha was indeed numerically stronger in the hand next condition compared to the hand under condition, but the direct comparison between the two suggests a rather comparable reduction in parietal alpha and provided no evidence for a lateralization of this. While these results do not rule out a contribution of spatial attention to the reduction in alpha power associated with the illusion, this cannot be the only contributing factor.

Beta band activity has been associated with the preparation of motor acts and sensorimotor feedback mechanisms for monitoring the status quo or planning future actions [18, 20, 73]. In fact, reduced beta power is observed during movement preparation and the intention to move. One study has shown that the rubber hand illusion maybe accompanied by small involuntary movements of the hand [7]. Hence, the lateralized reduction in beta power that we observed around illusion onset may possibly arise from micro-movements associated with experiencing the illusory feeling. Related to the notion of motor planning, beta band activity has also been implied in predictive coding and the processing of prediction errors [73–75]. Here, stronger beta may have been associated with a higher precision of internal predictions, suggesting that the reduction of beta power may reflect a reduced precision of how well the external world can be predicted during the experienced discrepancy between visual and tactile information during the illusory state. Furthermore, the sensory-motor information during the control condition may be better predicted by internal models, since this condition matches the expected model of the reality, while the Illusion may diverge from these internal predictions. In the present data we observed a strong and lateralized reduction in beta power around the illusion onset. The reduction of beta over parietal electrodes was stronger over the right hemisphere while the reduction over central electrodes was stronger over the left. Hence, the observed reduction in beta may reflect multiple processes, comprising effects related to motor feedback but also to the overall excitability of the sensorimotor system in general. Studies with suitable control conditions are required to more precisely disentangle these distinct processes in the future.

We also note that the illusory state is accompanied by an increase in local gamma band activity in posterior parietal cortex and premotor cortex after illusion onset [11]. Opposing changes in low and high-frequency rhythmic brain activity may reflect the differential engagement of feed-forward and feed-back processes [74, 76, 77]. While gamma band activity has been often associated with the processing of feed-forward signals, alpha and beta band activity has been associated with top-down processes or the modulation of feed-forward pathways, as discussed above. Collectively, correlates of the illusory state in low and high frequency brain activity likely reflect the multitude of processes underlying the illusory state, which have also been highlighted by a number of fMRI studies: these include the bottom-up integration of visual-tactile signals in parietal regions [1], changes in bodily self-awareness in parietal and premotor regions [22], and possibly the modulation of bottom-up signals following a change in the perceived body ownership [74, 76, 77], as well as a general increase in sensorimotor activation as result of the illusory feeling [5, 15–17].

## General conclusion

The question of which neurophysiological marker of brain activity may be specifically related to the subjective illusory state during the rubber hand illusion remains difficult to address. Brain activity related to this illusory state is usually isolated by contrasting data obtained during the illusion with data obtained in suitable control conditions. However, the widely used control conditions for the rubber hand differ from the illusion configuration in more than one factor, including the rotation of the rubber hand, they may involve the absence of this, they may rely on a different temporal pattern of visuo-tactile stimulation or may differ by demand characteristics. As a result, any difference observed between illusion epochs and each individual control condition cannot be attributed to a single factor, such as the illusory state. To address this conundrum, we employed multiple contrasts to pinpoint correlates that robustly emerge across multiple comparisons between conditions. The collective results suggest that a reduction in alpha and beta power accompanies the illusion across multiple control conditions, and point in particular to changes in beta activity around the moment of the illusion onset.

## Supporting information

S1 Appendix Figures scripts. (ZIP)
